# Evaluation of an automated connective tissue disease screening assay in Korean patients with systemic rheumatic diseases

**DOI:** 10.1371/journal.pone.0173597

**Published:** 2017-03-08

**Authors:** Seri Jeong, Heeyoung Yang, Hyunyong Hwang

**Affiliations:** 1 Department of Laboratory Medicine, Kosin University College of Medicine, Busan, Republic of Korea; 2 Department of Laboratory Medicine, Gyeonggi Provincial Medical Center Paju Hospital, Gyeonggi, Republic of Korea; Peking University First Hospital, CHINA

## Abstract

This study aimed to evaluate the diagnostic utilities of the automated connective tissues disease screening assay, CTD screen, in patients with systemic rheumatic diseases. A total of 1093 serum samples were assayed using CTD screen and indirect immunofluorescent (IIF) methods. Among them, 162 were diagnosed with systemic rheumatic disease, including rheumatoid arthritis (RA), systemic lupus erythematosus (SLE), and mixed connective tissue disease (MCT). The remaining 931 with non-systemic rheumatic disease were assigned to the control group. The median ratios of CTD screen tests were significantly higher in the systemic rheumatic disease group than in the control group. The positive likelihood ratios of the CTD screen were higher than those of IIF in patients with total rheumatic diseases (4.1 vs. 1.6), including SLE (24.3 vs. 10.7). The areas under the receiver operating characteristic curves (ROC-AUCs) of the CTD screen for discriminating total rheumatic diseases, RA, SLE, and MCT from controls were 0.68, 0.56, 0.92 and 0.80, respectively. The ROC-AUCs of the combinations with IIF were significantly higher in patients with total rheumatic diseases (0.72) and MCT (0.85) than in those of the CTD screen alone. Multivariate analysis indicated that both the CTD screen and IIF were independent variables for predicting systemic rheumatic disease. CTD screen alone and in combination with IIF were a valuable diagnostic tool for predicting systemic rheumatic diseases, particularly for SLE.

## Introduction

Patients with systemic rheumatic diseases, including rheumatoid arthritis (RA), systemic lupus erythematosus (SLE), mixed connective tissue disease (MCT), Sjögren’s syndrome, and systemic sclerosis, commonly suffer from diffuse organ damage, associated with auto-antibodies [1]. Anti-nuclear antibodies (ANA), a kind of auto-antibodies, are directed against a variety of nuclear antigens. The detection of ANA was reported to be useful for diagnosis of patients with systemic rheumatic diseases [2,3]. Indirect immunofluorescence (IIF) assay on cultured human epithelial carcinoma cells (HEp-2 cell) has been used as a gold standard method. However, IIF is a time-consuming and labor-intensive procedure and exhibits poor reproducibility because of the subjective interpretation of results [3,4].

Enzyme immunoassays (EIA) have been developed for ANA screening instead of IIF and are widely used in clinical laboratories [5]. The CTD screen (Thermo Fisher Scientific Inc.; Freiburg, Germany) used in this study is a recently introduced EIA-based assay with 17 different human recombinant antigens. Commercially available EIA kits allow automatization and quantification of ANA screening. A few reports have evaluated the performance of the EIA kits, which were similar to the CTD screen [6–8]. The previously reported EliA Symphony (Pharmacia Diagnostics, Freiburg, Germany) detects antibodies to 9 antigens, including SSA/Ro, SSB/La, U1RNP (RNP70, A, C), Scl-70, Jo-1, Centromere B, and Sm [6]. The 17 antigens of the Phadia EliA CTD screen (Phadia AB, Freiburg, Germany and Phadia AB, Uppsala, Sweden), described by Op De Beeck et al. [7] and Parker et al. [8], were not different from those in the CTD screen in our study. The study populations of these reports were patients in hospitals of Spain, Belgium, and the United Kingdom in Europe. No reports have examined Korean patients as a study population to assess the results of a CTD screen and IIF combination.

In this study, we evaluated diagnostic values of an automated connective tissue disease screening assay, CTD screen, in patients with systemic rheumatic disease. Diagnostic performance of the assay was compared with that of HEp-2 cell-based IIF in a large Korean population. We also investigated the diagnostic performance of a combination of the CTD screen and IIF for each systemic rheumatic disease.

## Materials and methods

### Ethics statement

This study was exempted from informed consent by the independent Institutional Review Board of Kosin University Gospel Hospital (Approval number: KUGH MDIRB 10–49).

### Study design

A total of 1093 serum samples from patients who visited Kosin University Gospel Hospital for systemic rheumatic disease evaluation were collected to demonstrate the diagnostic performance of CTD screen (Thermo Fisher Scientific Inc.). The specimens were randomly collected, and the results of same patients were not included repeatedly in the data set of our study. The minimal data set of this study is provided in S1 File. The specimens were classified according to the predefined patient diagnosis as follows: Total systemic rheumatic disease (n = 162), RA (n = 100), SLE (n = 35), MCT (n = 23), Sjögren’s syndrome (n = 2), systemic sclerosis (n = 2), and control (n = 931). The total systemic rheumatic disease group was composed of patients with RA, SLE, MCT, Sjögren’s syndrome, and systemic sclerosis. Although Sjögren’s syndrome and systemic sclerosis were included in the total systemic rheumatic disease group, they were not grouped separately due to their paucity in order to allow statistical analysis. It was impossible to present the median values of age and CTD screen ratio in the patients with Sjögren’s syndrome and systemic sclerosis because the data must contain at least 3 observations in order to calculate the median values. Further, the 95% confidence intervals (CIs) of the odds ratios in multivariate logistic regression analysis were not generated by the statistical programs in this study. Therefore, the data of the patients with Sjögren’s syndrome and systemic sclerosis were included and reflected in the total systemic rheumatic disease group. Additionally, the group for total rheumatic diseases without RA was also analyzed because the ANA was not closely related to RA.

All patients were diagnosed by specialized rheumatologists in the clinics of Kosin University Gospel Hospital based on the criteria of the American College of Rheumatology/European League and American Rheumatism Association [9,10] for RA, the American College of Rheumatology and Systemic Lupus International Collaborating Clinics [11,12] for SLE, the American College of Rheumatology/European League [13] for systemic sclerosis, Alarcón-Segovia et al. [14] for MCT, and the American-European consensus classification [15] for Sjögren’s syndrome. The control group was composed of patients with several non-systemic rheumatic diseases, reflecting actual clinical laboratory conditions. All of the collected serum samples were frozen in aliquots at or below -20℃ until assayed, and only one freeze/thaw cycle was conducted for each assay.

### Indirect immunofluorescence for anti-nuclear antibody

Indirect immunofluorescence microscopy on HEp-2 cells for anti-nuclear antibody was conducted with a commercially available kit (Fluoro HEPANA test, MBL Co., LTD, Nagoya, Japan), according to the manufacturer’s instructions. The serum samples were diluted with phosphate buffered saline to a 1:20 ratio for qualitative analysis. One drop (30–40 μL) of each diluted serum was applied to the slides on which HEp-2 cells were fixed, and the slides were incubated in the moisture chamber for 20 minutes at room temperature (20–25℃). After rinsing, the secondary reaction was performed with FITC-conjugated goat anti-human immunoglobulin antibody, followed by a washing step. Mounted slides were examined with fluorescent microscopy at 400x magnification. Positive and negative controls were used for quality control. The threshold for positivity was a titer of 1:20, which was suggested by the manufacturer and is routinely applied in the clinical laboratory.

### CTD screen

The EliA^TM^ CTD screen (Thermo Fisher Scientific Inc.), a fluorescence enzyme immunoassay, was performed on the Phadia 250 instrument (Thermo Fisher Scientific Inc.) to detect antibodies to nuclear target antigens. The 17 antigens coated with the EliA CTD screen, indicated in the instructions of the manufacturer, are presented in S1 Table. The assays were conducted according to the manufacturer’s instructions, similar to the procedures of a previous report [8]. Results were calculated automatically by the instrument. A ratio of tested serum sample response to a calibrator greater than 1.0 was positive, 0.7 to 1.0 was equivocal, and less than 0.7 was negative.

### Statistical analysis

Statistical analyses were performed using PASW version 24.0 (formerly, SPSS Statistics) (SPSS Inc., Chicago, IL, USA) and Analyse-it Method Evaluation Edition, version 2.26 software (Analyse-it Software Ltd., Leeds, UK). Comparisons of nominal variables and continuous variables between two groups were assessed by Fisher’s exact test and Mann-Whitney *U* test, respectively. Multiple comparisons among the subgroups, including RA, SLE, MCT and control, were performed using the Chi-square test and Kruskal-Wallis test with pairwise comparison and Bonferroni correction to compensate for alpha statistical errors. Kappa coefficients were calculated to estimate the agreement between the results of CTD screen and IIF. Receiver operating characteristic (ROC) curves were plotted for CTD screen and its combination with IIF in order to assess their diagnostic abilities to differentiate between systemic rheumatic diseases and the control group. The areas under the ROC curves (AUCs) of CTD screen and its combination with IIF were compared. Binary logistic regression analysis was performed to calculate the predicted probability values of the IIF and CTD screen combinations, and these values were used to estimate the ROC-AUCs for IIF and CTD screen combinations, similar to the previously described method [16]. The presence of systemic rheumatic disease was the dependent variable, and the results of IIF and CTD screen were identified as the co-variables. Multivariate logistic regression analysis was performed with the presence of systemic rheumatic diseases as the dependent variable and age, sex, and the results of IIF and CTD screen as co-variables. *P-*values less than 0.05 were considered statistically significant.

## Results

### Study population characteristics

The basic characteristics of the study population are shown in Table 1. The proportion of female patients was higher in the total systemic rheumatic disease group than in the control group (80.2% vs. 55.1%, *P*<0.0001). Female patients were statistically predominant in the total disease without RA (95.2%, *P*<0.0001), RA (71.0%, *P =* 0.0028), SLE (91.4%, *P*<0.0001), and MCT (100.0%, *P*<0.0001) groups. The median ages of the patients in the total disease and control groups were 45.0 and 50.0 years, respectively (*P =* 0.0007). The median age difference between the RA and control group was not significant (49.0 vs. 50.0, *P =* 1.0000), while the total disease without RA (39.5, *P*<0.0001), SLE (39.0, *P*<0.0001) and MCT (43.0, *P =* 0.0134) groups showed significant differences (Table 1 and S2 Table).

**Table 1:** Study population characteristics and IIF and CTD screen results based on study group.

Parameters		Predefined rheumatic diseases					Control (n = 931)	*P* value^a^
		Total disease (n = 162)	Total disease without RA (n = 62)	RA (n = 100)	SLE (n = 35)	MCT (n = 23)		
No. of females, %		130, 80.2	59, 95.2	71, 71.0	32, 91.4	23, 100.0	513, 55.1	<0.0001
Age (years)^b^		45.0 (34.0–53.1)	39.5 (18.0–50.1)	49.0 (38.8–55.0)	39.0 (14.0–44.8)	43.0 (31.0–43.8)	50.0 (37.0–62.0)	0.0007
IIF (No.), %	Positive	100, 61.7	55, 88.7	45, 45.0	32, 91.4	19, 82.6	227, 24.4	<0.0001
	Negative	62, 38.3	7, 11.3	55, 55.0	3, 8.6	4, 17.4	704, 75.6	
IIF pattern (No.), %	Homogeneous	43, 26.5	22, 35.5	21, 21.0	15, 42.9	5, 21.7	105, 11.3	0.1622
	Speckled	23, 14.2	15, 24.2	8, 8.0	8, 22.9	7, 30.4	41, 4.4	
	Centromere	5, 3.1	4, 6.5	1, 1.0	0, 0.0	3, 13.0	3, 0.3	
	Nucleolar	4, 2.5	0, 0.0	4, 4.0	0, 0.0	0, 0.0	16, 1.7	
	Cytoplasmic	13, 8.0	6, 9.7	7, 7.0	5, 14.3	1, 4.3	42, 4.5	
	Other	12, 7.4	8, 12.9	4, 4.0	4, 11.4	3, 13.0	20, 2.1	
CTD screen (ratio)^b^		0.3 (0.1–2.3)	4.1 (0.3–10.1)	0.2 (0.1–0.4)	7.1 (2.7–12.0)	1.1 (0.2–4.0)	0.1 (0.1–0.2)	<0.0001

IIF, indirect immunofluorescence; RA, rheumatoid arthritis; SLE, systemic lupus erythematosus; MCT, mixed connective tissue disease.

^a^ Fisher's exact test for nominal variables and Mann-Whitney *U* test for continuous variables of the total disease vs. control.

^b^ Data are expressed as median (1st to 3rd quartiles).

### IIF and CTD screen comparisons according to predefined rheumatic diseases

**Fig 1:**
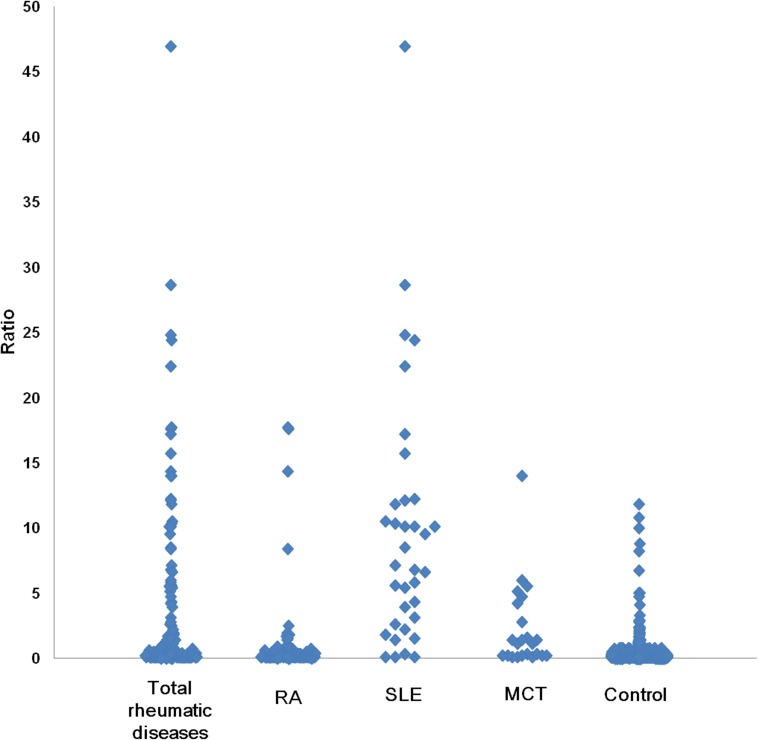
CTD screen reactivity in patients with systemic rheumatic diseases compared with the control group. The highest CTD screen reactivity was found in patients with systemic lupus erythematosus (SLE), whereas the lowest reactivity was found in the control group. Abbreviations: IIF, indirect immunofluorescence; RA, rheumatoid arthritis; SLE, systemic lupus erythematosus; MCT, mixed connective tissue disease.

The results of the IIF and CTD screen in patients with predefined rheumatic diseases and in the control group are presented in Table 1 and Fig 1. The results show that IIF was positively associated with the total disease, total disease without RA, RA, SLE and MCT groups (*P*<0.0001 for all). The categorized IIF patterns were not significantly associated with the total rheumatic disease group compared to the control group (*P =* 0.1622). The median CTD screen ratios in all of the predefined rheumatic disease groups (total disease (0.3, *P*<0.0001), total disease without RA (4.1, *P*<0.0001), SLE (7.1, *P*<0.0001) and MCT (1.1, *P*<0.0001) groups) were significantly higher than that in control group, with the exception of the RA (0.2, *P =* 0.2371) group (Table 1 and S2 Table). In addition, the stratified study population characteristics and the results of IIF and CTD screen in the sex- and age-matched controls are presented in the S3 Table to investigate the influence of sex and age on the results of IIF and CTD screen.

### Overall IIF and CTD screen agreement

The results of the CTD screen compared to IIF are summarized in Table 2. When CTD screen ratios were considered as qualitative results with cut-offs, as suggested by the manufacturer, the overall concordance rates between IIF and the CTD screen were 76.7% (cut-off 1.0; equivocal ratios as negative results) and 76.6% (cut-off 0.7; equivocal ratios as positive results). The kappa coefficients between the two assays were fair (0.31 for cut-off 1.0 and 0.33 for cut-off 0.7). The median level of CTD screen ratio was higher in the positive IIF group than that in the negative IIF group (*P*<0.0001).

**Table 2:** Qualitative and quantitative CTD screen results compared to IIF (n = 1093).

Qualitative	Agreement	IIF		*P* value^a^
**CTD screen**	kappa^b^ = 0.31 (0.25–0.37)	Positive (%)	Negative (%)	<0.0001
Cut-off 1.0	Positive (%)	90 (8.2)	18 (1.6)	
	Negative (%)	237 (21.7)	748 (68.4)	
Cut-off 0.7	kappa^b^ = 0.33 (0.27–0.38)	Positive (%)	Negative (%)	<0.0001
	Positive (%)	101 (9.2)	30 (2.7)	
	Negative (%)	226 (20.7)	736 (67.3)	
Quantitative (ratio)^c^	-	0.24 (0.13–1.30)	0.13 (0.09–0.21)	<0.0001

IIF, indirect immunofluorescence.

^a^ Fisher's exact test for nominal variables and Mann-Whitney *U* test for continuous variables.

^b^ Shown as value (95% confidence interval).

^c^ Data of CTD screen, ratios of tested serum samples response to a calibrator, are expressed as median (1st to 3rd quartiles).

### CTD screen diagnostic performances

**Fig 2:**
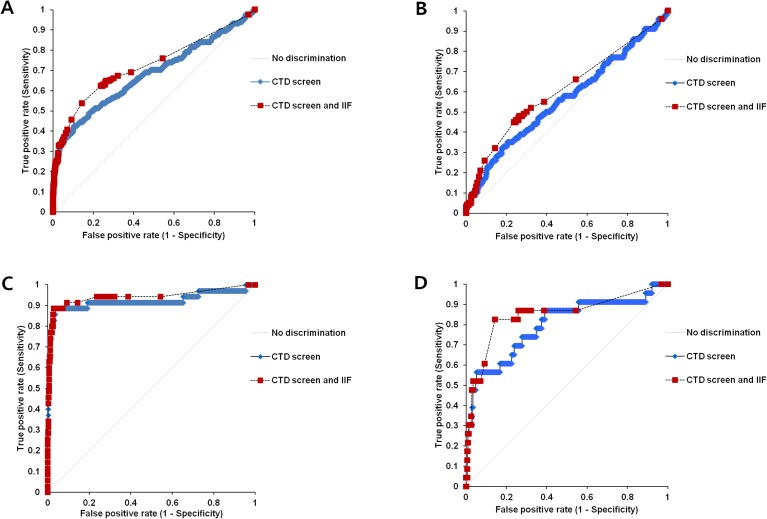
Diagnostic performance of CTD screen and its combination with IIF for predicting systematic rheumatic diseases. (A) Receiver operating characteristic (ROC) curves of CTD screen and its combination with indirect immunofluorescence (IIF) for discriminating total systemic rheumatic disease (n = 162) from the control group (n = 931). The areas under the ROC curves (AUCs) for CTD screen and its combination with IIF were 0.68 and 0.72, respectively, demonstrating a significant difference (*P* = 0.0054). (B) ROC curves for CTD screen and its combination with IIF for differentiating rheumatoid arthritis (RA) (n = 100) from the control group. The AUCs of CTD screen and its combination with IIF were 0.56 and 0.61, respectively. (C) ROC curves for the CTD screen combined with IIF for differentiating systemic lupus erythematosus (SLE) (n = 35) from the control group. The AUCs of CTD screen and its combination with IIF were 0.92 and 0.94, respectively. (D) ROC curves for CTD screen and its combination with IIF for discriminating mixed connective tissue disease (MCT) (n = 23) from the control group. The AUCs of CTD screen and its combination with IIF were 0.80 and 0.85, respectively, and they were statistically different (*P* = 0.0410).

Fig 2 displays the ROC curves of the CTD screen for discriminating cases with systemic rheumatic diseases from those without. The AUC of the CTD screen for differentiating total systemic rheumatic diseases (n = 162) from all other conditions (n = 931) was 0.68 (Fig 2A), and that for distinguishing RA (n = 100), SLE (n = 35), and MCT (n = 23) from control subjects was 0.56, 0.92, and 0.80, respectively (Fig 2B–2D). We also analyzed the AUCs of IIF and CTD screen combinations when differentiating systemic rheumatic diseases from the control group. Total systemic rheumatic diseases (0.68 vs. 0.72, *P* = 0.0054) and MCT (0.80 vs. 0.85, *P* = 0.0410) groups showed significant differences between the AUCs of the CTD screen and those combined with IIF, while RA (0.56 vs. 0.61, *P* = 0.0531) and SLE (0.92 vs. 0.94, *P* = 0.1357) groups did not.

The resulting ROC-AUCs, sensitivities, specificities at the best cut-offs, and likelihood ratios are summarized in Table 3. The best cut-off was determined when the sum of sensitivity and specificity was maximized. When these cut-off values were applied, the sensitivities of the CTD screen and its combination with IIF were 42.6% and 53.7%, respectively, and the specificities were 89.5% and 85.7%. The sensitivities and specificities of excellent or good AUCs in each group were as follows: The total rheumatic diseases without RA group (n = 62), 74.2% and 94.7% for the CTD screen and 88.7% and 85.7% for combination with IIF, respectively; SLE group (n = 35), 88.6% and 96.3 for the CTD screen and 88.6% and 97.1% for combination with IIF; MCT group (n = 23), 56.5% and 94.7% for the CTD screen and 82.6% and 85.7% for combination with IIF. The ROC-AUCs of the CTD screen for discriminating IIF positivity from negativity ranged from 0.66 to 0.88.

**Table 3:** Sensitivity, specificity, and ROC-AUC of CTD screen, tested independently and in combination^a^.

Predefined diseases	Parameter	CTD screen	CTD screen + IIF	CTD screen vs. IIF
Total rheumatic diseases (n = 162)	ROC-AUC ^b^	0.68 (0.63–0.73)	0.72 (0.67–0.77)	0.71 (0.67–0.74)
	Sensitivity (%)^b^	42.6 (34.9–50.6)	53.7 (45.7–61.6)	48.3 (42.8–53.9)
	Specificity (%)^b^	89.5 (87.3–91.4)	85.7 (83.3–87.9)	83.9 (81.1–86.5)
	+LR	4.1	3.8	3.0
	-LR	0.6	0.5	0.6
Total rheumatic diseases without RA (n = 62)	ROC-AUC ^b^	0.87 (0.80–0.93)	0.91 (0.86–0.96)	0.88 (0.69–1.00)
	Sensitivity (%)^b^	74.2 (61.5–84.5)	88.7 (78.1–95.3)	98.2 (90.3–100.0)
	Specificity (%)^b^	94.7 (93.1–96.1)	85.7 (83.3–87.9)	71.4 (29.0–96.3)
	+LR	14.1	6.2	3.4
	-LR	0.3	0.1	<0.1
RA (n = 100)	ROC-AUC ^b^	0.56 (0.50–0.63)	0.64 (0.55–0.67)	0.66 (0.62–0.70)
	Sensitivity (%)^b^	35.0 (25.7–45.2)	48.0 (37.9–58.2)	41.2 (35.3–47.3)
	Specificity (%)^b^	79.5 (76.7–82.0)	73.7 (70.7–76.5)	84.1 (81.3–86.6)
	+LR	1.7	1.8	2.6
	-LR	0.8	0.7	0.7
SLE (n = 35)	ROC-AUC ^b^	0.92 (0.85–1.00)	0.94 (0.88–1.00)	0.69 (0.65–0.73)
	Sensitivity (%)^b^	88.6 (73.3–96.8)	88.6 (73.3–96.8)	45.2 (39.0–51.5)
	Specificity (%)^b^	96.3 (94.9–97.5)	97.1 (95.8–98.1)	84.0 (81.1–86.6)
	+LR	24.3	30.5	2.8
	-LR	0.1	0.1	0.6
MCT (n = 23)	ROC-AUC ^b^	0.80 (0.68–0.91)	0.85 (0.75–0.95)	0.67 (0.63–0.71)
	Sensitivity (%)^b^	56.5 (34.5–76.8)	82.6 (61.2–95.0)	40.2 (34.1–46.7)
	Specificity (%)^b^	94.7 (93.1–96.1)	85.7 (83.3–87.9)	84.0 (81.1–86.7)
	+LR	10.7	5.8	2.5
	-LR	0.5	0.2	0.7

ROC-AUCs, areas under the receiver operating characteristic curves; IIF, indirect immunofluorescence; RA, rheumatoid arthritis; SLE, systemic lupus erythematosus; MCT, mixed connective tissue disease; LR, likelihood ratio.

^a^ Fisher's exact test for nominal variables and Mann-Whitney *U* test for continuous variables.

^b^ Data are shown as value (95% confidence interval).

The positive likelihood ratios with conclusive or moderate changes in probability were seen with the CTD screen and its combination with IIF in total rheumatic diseases without RA (14.1 and 6.2, respectively), SLE (24.3 and 30.5, respectively) and MCT (10.7 and 5.8, respectively) groups, according to the criteria suggested by the “Evidence-based Medicine Working Group” [17]. The negative likelihood ratios of each studied methods, considered to be very useful based on the criteria of Solomon et al., were those of CTD screen and its combination with IIF in SLE (0.1 for all) and those of combination with IIF (0.1) and CTD screen vs. IIF (<0.1) in total rheumatic diseases without RA [3].

### Multivariate analysis of IIF and CTD screen

Multivariate analysis was performed with the presence of systemic rheumatic diseases as the binary dependent variable and the age and sex of the patient as well as the IIF and CTD screen results as predictors. We found sex, IIF, and CTD screen to be independently associated with total systemic rheumatic disease (Table 4). Based on our results, age, sex, IIF and CTD screen for total rheumatic diseases without RA; sex and IIF for RA; age, IIF, and CTD screen for SLE; and age, IIF, and CTD screen for MCT were independently related with the respective rheumatic disease. Sex was not a predictor in the MCT group in the multivariate analysis because all of the subjects were female.

**Table 4:** Multivariate analysis^a^ of the outcomes of systematic rheumatic disease.

Dependent variables	Covariate	OR	SE	95% CI	*P* value
Total rheumatic diseases (n = 162)	Age (years)	0.9906	0.0054	0.9802 to 1.0011	0.0799
	Male sex	0.4250	0.2208	0.2757 to 0.6553	0.0001
	IIF	2.9232	0.1999	1.9757 to 4.3251	<0.0001
	CTD screen (ratio)	1.3265	0.0544	1.1923 to 1.4759	<0.0001
Total rheumatic diseases without RA (n = 62)	Age (years)	0.9676	0.0100	0.9489 to 0.9867	0.0009
	Male sex	0.1272	0.6495	0.0356 to 0.4544	0.0015
	IIF	9.1079	0.4471	3.7918 to 21.8772	<0.0001
	CTD screen (ratio)	1.5013	0.0683	1.3133 to 1.7162	<0.0001
RA (n = 100)	Age (years)	0.9976	0.0061	0.9857 to 1.0096	0.6917
	Male sex	0.5519	0.2336	0.3492 to 0.8723	0.0109
	IIF	2.1416	0.2270	1.3726 to 3.3416	0.0008
	CTD screen (ratio)	1.1359	0.0567	1.0165 to 1.2693	0.0245
SLE (n = 35)	Age (years)	0.9604	0.0140	0.9344 to 0.9872	0.0040
	Male sex	0.3739	0.7220	0.0908 to 1.5392	0.1730
	IIF	0.0035	0.6930	1.9491 to 29.4843	0.0035
	CTD screen (ratio)	1.6381	0.0790	1.4031 to 1.9126	<0.0001
MCT^b^ (n = 23)	Age (years)	0.9634	0.0133	0.9387 to 0.9888	0.0050
	IIF	9.3781	0.5849	2.9802 to 29.5104	0.0001
	CTD screen (ratio)	1.2869	0.0888	1.0814 to 1.5315	0.0045

OR, odds ratio; SE, standard error; CI, confidence interval; IIF, indirect immunofluorescence; RA, rheumatoid arthritis; SLE, systemic lupus erythematosus; MCT, mixed connective tissue disease.

^a^ Multivariate analysis was performed with the presence of each systemic rheumatic disease as binary dependent variables and with the age and sex of patients and the results of IIF and CTD screen as covariates.

^b^ The MCT group was composed of only female.

## Discussion

Diagnostic applications of CTD screen, an automated screening assay with 17 antigens, were evaluated in Korean patients with systemic rheumatic diseases. The assay performance was compared to HEp-2 cell-based IIF and diagnostic values of combined CTD screen and IIF for each systemic rheumatic disease.

Although anti-nuclear antibodies can be found in patients with non-rheumatic diseases and even in healthy individuals [3], the proportion of IIF positivity and the median level of CTD screen were higher in the predefined rheumatic diseases than in the control group (*P*<0.0001 for all, except CTD screen in RA). Previous studies have also reported similar results of IIF and CTD screen [6,7]. The highest reactivity of CTD screen was found in patients with SLE, followed by those with MCT and RA. Although there were 3 SLE and 4 MCT patients with negative IIF results, they showed earlier clinical observations with developed positive IIF later in follow-up or negative conversion after initial positivity on presentation because of immunosuppressive treatment according to the review of medical records. Several studies described the variable clinical and serological presentations over time in patients with SLE and MCT [18,19].

The classic patterns of IIF with systemic rheumatic disease were generally concordant with our results, considering the proportion of each value of IIF pattern. Homogeneous staining includes anti-dsDNA, which showed high specificity for SLE [20]. Speckled nucleoplasmic staining covers anti-Sm and anti-nRNP. Anti-Sm auto-antibodies were pathognomonic of SLE, with SLE occurring within one year when the antibodies were detected in asymptomatic patients [21]. The sensitivity and specificity of anti-Sm were 24% and 98% in patients with SLE according to the random-effect model, respectively [22]. Anti-nRNPs, auto-antibodies for the U1, U2, U4-6, and U5 small nuclear ribonucleoproteins, showed 71–100% sensitivity and 84–100% specificity in patients with MCT [22]. Centromere and nucleolar patterns of IIF, which were primarily associated with systemic sclerosis, were found in the control group, because the control group consisted of patients with several non-systemic rheumatic diseases such as arthritis, synovitis, hemangioma, urticaria, dermatitis, allergic rhinitis, hepatic diseases, chronic renal insufficiency, cancer, gastrointestinal diseases, sarcoidosis and hypertension. According to previous reports, anti-centromere antibodies were found in patients with hepatitis and asymptomatic individuals with rheumatic diseases [23] and anti-RNA polymerase and anti-PM/Scl antibodies, related to nucleolar pattern, were detected in cancer cells and even in the general population [24,25].

The agreement between IIF and CTD screen was fair, according to the Kappa coefficient classification [26], which indicates whether the equivocal results of the CTD screen were positive or negative. IIF had higher positivity than the qualitative CTD screen, regardless of the cut-offs in the studied patients with systemic rheumatic diseases. The proportions of IIF positive and CTD screen negative results at the cut-off 1.0 and 0.7 were 21.7% and 20.7%, respectively, while those of IIF negative and CTD screen positive results at the cut-offs of 1.0 and 0.7 were 1.6% and 2.7%, respectively. Solid-phase assays have been reported to exhibit lower sensitivity than the IIF method, and a case report has indicated that the solid-phase assay resulted in delay of SLE diagnosis due to a false negative ANA test [5,27]. Based on medical records, patients in an inactive or mildly active phase of rheumatic disease showed the discrepancies between IIF and CTD screen. The scores of Systemic Lupus Erythematosus Disease Activity Index (SLEDAI) in these patients ranged from 0 to 5. Additionally, previous studies have reported that solid-phase assays were not able to identify auto-antibodies to missed extractable nuclear antigens [28,29]. The CTD screen was reported to be insufficient in screening for rare antibodies such as anti-RNA polymerase III and anti-fibrillarin [8].

In our data, the sensitivities of IIF were higher than those of the CTD screen (61.7% vs. 42.6% for total rheumatic disease; 88.7% vs. 74.2% for total rheumatic disease without RA; 45.0% vs. 35.0% for RA; 91.4% vs. 88.6% for SLE; 82.6% vs. 56.5% for MCT), whereas the specificities of the CTD screen were higher than those of IIF (89.5% vs. 80.0% for total rheumatic diseases) in all of the studied groups. These trends of sensitivity and specificity of solid-phase assays and IIF were concordant to previously mentioned reports. The sensitivity of IIF was 91%, while that of solid-phase assays ranged from 49% to 89% according to Bernardini et al. [30] and Bonilla et al. [31]. González et al. [6] showed that the sensitivity and specificity of IIF were 69% and 89%, while those of EliA Symphony were 63% and 91%, respectively. The slight differences in sensitivity and specificity might be caused by different populations and IIF titer cut-offs among the studies.

The positive likelihood ratios of CTD screen were higher than those of IIF in all studied groups (total rheumatic diseases, 4.1 vs. 1.6; total rheumatic diseases without RA, 14.1 vs. 7.9; RA, 1.7 vs. 0.8; SLE, 24.3 vs. 10.7; MCT, 10.7 vs. 4.8). Therefore, the post-test probability for each systemic rheumatic disease was higher for a positive CTD screen result than for a positive IIF result. The positive likelihood ratios of CTD screen for patients with SLE and MCT were particularly increased, indicating conclusive changes in pre-test to post-test probability [32]. Op De Beeck et al. [7] also reported that a positive result on CTD screen had a higher likelihood ratio than a positive result by IIF. On the other hand, the negative likelihood ratios of IIF were lower than those of CTD screen with the exception of SLE patients (0.5 vs. 0.6 for total rheumatic diseases; 0.1 vs. 0.3 for total rheumatic diseases without RA; 0.7 vs. 0.8 for RA; 0.1 vs. 0.1 for SLE; 0.2 vs. 0.5 for MCT). CTD screen in patients with SLE and IIF with SLE and MCT were considered to be useful test based on the negative likelihood ratios of the two methods. Lower likelihood ratios for a negative test result with IIF compared to those for a negative result with CTD screen were also observed in a previous study [7].

The diagnostic abilities of CTD screen to differentiate systemic rheumatic diseases from the control group were analyzed because the clinical utility of CTD screen can vary with the characteristics of systemic rheumatic patients and non-rheumatic conditions. As a result, CTD screen showed the highest ROC-AUC of 0.92 in patients with SLE, followed by 0.87 in patients with total rheumatic diseases without RA, and 0.80 in patients with MCT. On the other hand, the AUCs of the CTD screen with total rheumatic diseases and RA were 0.68 and 0.56 for discriminating rheumatic diseases from the control group, respectively. The considerable portion of RA patients in the total rheumatic diseases group could be a reason for the poor CTD screen performance in total rheumatic diseases based on the AUCs, compared to the AUCs of CTD screen in the total disease without RA group. A previous study that excluded RA patients from the study population showed a 0.823 AUC with CTD screen [6].

In our results, simultaneous determination of CTD screen and IIF demonstrated an improved diagnostic utility in patients with total systemic rheumatic diseases (0.72 vs. 0.68, *P* = 0.0054), total rheumatic diseases without RA (0.91 vs. 0.87, *P* = 0.0033), and MCT (0.85 vs. 0.80, *P* = 0.0410) compared to the CTD screen alone. Additionally, using only CTD screen was effective for patients with SLE (0.94 for combination and 0.92 for CTD alone, *P* = 0.1357). The diagnostic performances of CTD screen and the combination of CTD screen and IIF were excellent in the total rheumatic diseases without RA group and SLE group and good in the MCT group, based on the AUCs [33]. Therefore, concurrent screening by CTD screen and IIF in patients with systemic rheumatic diseases, especially in cases of strong clinical impression of MCT, would be useful. Furthermore, investigation of laboratory findings, which are referred to other immunologic disorders, might be essential for diagnosis when the screening assay showed positive results.

Multivariate analysis was also performed with age and sex in order to assess CTD screen and IIF as predictor variables for systemic rheumatic diseases and to control potential confounding factors. ANA was reported to be found in the healthy control group, especially women older than 40 years and elderly individuals [34]. Therefore, age and sex factors were incorporated into our multivariate model. The multivariate analysis results indicated that CTD screen and IIF were independently related to all of the studied systemic rheumatic diseases, with the exception of CTD screen in the RA group. Our result confirmed that screening for ANA testing is not preferable for ruling out RA; however, it can be applied to diagnoses of SLE and MCT [35,36].

Our study design has some limitation that most of our study population consisted of female. A recent study, reported hospitalization in SLE patients based on the results from the Korean lupus network registry, showed that 165 out of 180 patients (91.8%) were women [37]. Although our data reflected a predominant proportion of female patients with systemic rheumatic diseases in Korea, further studies that include male patients should be conducted. Moreover, the evaluation data of the CTD screen in larger numbers of study patients presenting with each systemic rheumatic disease would be necessary. In addition, the study, which performed quantitative analysis of IIF with serial dilutions, should be followed, because our study focused on the comparison between the qualitative results of IIF and CTD screen. The 1:20 ratio, used in our clinical laboratory in this study, could have the possibility of false positivity of IIF in control subjects.

## Conclusions

In conclusion, we evaluated the diagnostic utilities of CTD screen to predict systemic rheumatic diseases. Although there were a few published reports that discussed the usefulness of CTD screen, no report has assessed a large population of Korean patients in Asia or the diagnostic performance of the combined CTD screen and IIF for systemic rheumatic diseases. The CTD screen was excellent for patients with SLE, and the combination with IIF was more effective for the diagnosis of total systemic rheumatic diseases, including MCT than CTD screen alone. Our results provided recent information on the CTD screen and IIF methods for patients with systemic rheumatic diseases in order to facilitate appropriate patient managements with cautious clinical impressions of patients’ diagnosis.

## Supporting information

S1 File The minimal data set, including the study population characteristics and the results of IIF and CTD screen. (PDF)

S1 Table The 17 antigens^a^ coated with the EliA CTD screen. (DOC)

S2 Table The *P* values^a^ among variables of subgroups, including RA, SLE, MCT, and control. (DOC)

S3 Table The study population characteristics and the results of IIF and CTD screen in the sex- and age-matched controls. (DOC)
