# Supplementary material for: Evaluation of an automated connective tissue disease screening assay in Korean patients with systemic rheumatic diseases
Source: PLoS One. 2017 Mar 8;12(3):e0173597. doi: 10.1371/journal.pone.0173597 (PMC5342238; doi:10.1371/journal.pone.0173597)
Supplement: S1 File — (PDF) [file pone.0173597.s001.pdf]

S1 File. The minimal data set, including the study population characteristics and the results of IIF and CTD screen.

Age	Sex[a]	CTD screen	IIF[a]	Diagnosis[a]	SLE[a]	RA[a]	MCT[a]	IIF pattern
44	0	2.46	1	1		1		Cytoplasmic
40	1	1.06	1	0	0	0	0	Homogeneous
77	0	0.61	1	0	0	0	0	Cytoplasmic
39	1	0.09	0	0	0	0	0	
35	0	0.07	0	0	0	0	0	
56	0	0.12	0	0	0	0	0	
9	0	0.10	0	0	0	0	0	
51	0	0.24	0	0	0	0	0	
43	0	5.50	1	1			1	Centromere
85	0	6.69	1	0	0	0	0	Homogeneous
58	1	0.10	0	0	0	0	0	
47	1	0.05	0	1		1		
32	0	0.19	0	0	0	0	0	
52	1	0.04	0	0	0	0	0	
29	0	0.04	0	1		1		
34	1	0.06	0	0	0	0	0	
16	0	0.10	1	0	0	0	0	Nucleolar
26	0	0.06	0	0	0	0	0	
81	1	0.10	0	0	0	0	0	
77	0	14.00	1	1				Other
67	0	0.04	0	0	0	0	0	
73	1	0.23	1	0	0	0	0	Cytoplasmic
46	0	0.09	0	0	0	0	0	
40	0	0.18	0	0	0	0	0	
48	1	1.65	0	0	0	0	0	
43	1	0.12	1	0	0	0	0	Nucleolar

12	1	0.07	1	0	0	0	0	Homogeneous
54	0	0.07	0	1		1		
19	0	0.05	0	0	0	0	0	
55	0	0.86	1	0	0	0	0	Homogeneous
21	0	0.30	1	1	1			Homogeneous
63	1	0.07	0	0	0	0	0	
63	0	0.92	0	0	0	0	0	
33	1	0.05	0	0	0	0	0	
65	0	0.08	0	0	0	0	0	
37	0	0.09	0	0	0	0	0	
37	0	1.98	1	0	0	0	0	Other
73	0	0.13	1	0	0	0	0	Speckled
53	1	0.05	0	0	0	0	0	
38	1	0.13	0	0	0	0	0	
56	1	0.06	0	0	0	0	0	
56	0	1.40	1	1				Centromere
70	1	0.10	0	0	0	0	0	
2	1	0.23	0	0	0	0	0	
46	0	0.03	0	0	0	0	0	
44	0	0.07	1	0	0	0	0	Homogeneous
72	0	0.08	1	0	0	0	0	Homogeneous
55	0	0.05	0	0	0	0	0	
60	1	0.03	0	0	0	0	0	
47	0	0.11	0	0	0	0	0	
71	0	0.10	1	0	0	0	0	Homogeneous
59	1	0.03	0	1		1		
16	0	4.20	1	1			1	Homogeneous
66	0	0.08	0	0	0	0	0	

25	1	0.05	0	0	0	0	0	
54	0	0.14	0	0	0	0	0	
37	1	0.84	0	0	0	0	0	
37	1	0.16	0	0	0	0	0	
54	1	0.04	0	0	0	0	0	
56	1	0.13	0	0	0	0	0	
75	0	0.06	1	0	0	0	0	Homogeneous
72	1	0.04	1	0	0	0	0	Speckled
51	0	0.15	0	1		1		
43	0	1.40	1	1			1	Speckled
41	1	0.11	0	0	0	0	0	
20	0	22.42	1	1	1			Other
46	0	0.06	1	0	0	0	0	Cytoplasmic
44	0	0.13	1	0	0	0	0	Homogeneous
30	0	0.06	0	0	0	0	0	
47	0	0.29	0	0	0	0	0	
23	0	0.44	1	1		1		Homogeneous
63	0	0.04	1	0	0	0	0	Speckled
42	0	0.44	1	0	0	0	0	Nucleolar
8	0	0.11	1	0	0	0	0	Speckled
43	0	1.40	1	1			1	Speckled
10	1	0.66	0	0	0	0	0	
34	0	0.14	1	0	0	0	0	Cytoplasmic
50	0	0.33	0	0	0	0	0	
76	0	0.06	1	0	0	0	0	Homogeneous
31	0	0.15	0	0	0	0	0	
54	0	0.11	1	1		1		Speckled
68	1	0.15	1	0	0	0	0	Speckled

83	0	0.27	0	0	0	0	0	
82	1	0.32	0	0	0	0	0	
58	1	0.06	0	0	0	0	0	
40	0	14.00	1	1			1	Homogeneous
58	1	0.67	1	0	0	0	0	Cytoplasmic
48	1	1.67	0	0	0	0	0	
65	0	0.46	0	0	0	0	0	
27	1	0.07	0	0	0	0	0	
18	0	3.08	1	1	1			Homogeneous
59	0	8.76	1	0	0	0	0	Homogeneous
11	1	0.17	1	0	0	0	0	Other
55	1	0.08	0	0	0	0	0	
64	0	0.38	0	0	0	0	0	
83	0	0.16	0	0	0	0	0	
44	0	4.70	1	1			1	Speckled
62	0	0.16	0	0	0	0	0	
59	0	0.08	0	1		1		
50	1	0.14	0	0	0	0	0	
40	0	1.29	1	0	0	0	0	Homogeneous
65	0	0.11	0	0	0	0	0	
69	1	0.10	0	0	0	0	0	
44	1	0.13	0	0	0	0	0	
76	1	0.06	1	0	0	0	0	Homogeneous
41	0	0.07	0	0	0	0	0	
53	1	0.06	1	0	0	0	0	Homogeneous
41	0	6.00	1	1			1	Speckled
60	1	0.10	0	0	0	0	0	
52	0	0.03	1	0	0	0	0	Nucleolar

51	0	10.26	1	1	1			Speckled
10	0	0.16	1	0	0	0	0	Homogeneous
40	1	0.78	1	0	0	0	0	Homogeneous
43	0	0.07	0	0	0	0	0	
43	1	0.06	0	0	0	0	0	
37	0	0.26	0	0	0	0	0	
28	1	0.21	0	0	0	0	0	
20	0	0.10	0	0	0	0	0	
76	0	0.20	1	0	0	0	0	Speckled
46	1	0.04	0	0	0	0	0	
42	0	0.50	1	0	0	0	0	Nucleolar
50	1	0.11	0	0	0	0	0	
63	0	0.06	1	0	0	0	0	Speckled
60	0	0.39	0	0	0	0	0	
23	0	0.49	1	1		1		Homogeneous
47	0	0.55	1	1		1		Cytoplasmic
68	1	0.07	0	0	0	0	0	
61	0	0.25	0	0	0	0	0	
61	1	0.09	0	0	0	0	0	
42	0	8.40	1	1		1		Speckled
63	1	0.11	0	0	0	0	0	
42	0	0.10	0	0	0	0	0	
68	1	0.22	0	0	0	0	0	
70	0	0.37	0	0	0	0	0	
65	0	0.18	0	0	0	0	0	
54	0	0.22	0	0	0	0	0	
63	0	0.08	0	0	0	0	0	
66	0	0.14	1	0	0	0	0	Homogeneous

66	0	2.16	1	0	0	0	0	Other
60	0	0.04	0	0	0	0	0	
23	0	2.80	1	1			1	Cytoplasmic
46	0	0.57	0	0	0	0	0	
30	0	0.21	0	0	0	0	0	
44	0	0.09	0	0	0	0	0	
74	0	0.12	0	0	0	0	0	
66	0	0.12	1	0	0	0	0	Homogeneous
16	1	0.70	0	0	0	0	0	
23	0	0.09	1	0	0	0	0	Homogeneous
71	1	0.18	0	0	0	0	0	
52	1	0.06	0	0	0	0	0	
29	1	0.09	0	0	0	0	0	
43	0	0.10	1	0	0	0	0	Homogeneous
78	0	0.05	0	0	0	0	0	
48	1	0.18	0	1		1		
67	1	0.09	0	0	0	0	0	
56	1	0.06	0	0	0	0	0	
17	1	0.10	1	0	0	0	0	Homogeneous
38	1	0.09	0	0	0	0	0	
44	0	0.21	0	0	0	0	0	
28	1	0.07	0	0	0	0	0	
39	1	0.50	0	0	0	0	0	
22	1	0.12	0	0	0	0	0	
40	0	1.50	1	1		1		Homogeneous
75	1	0.27	0	0	0	0	0	
69	1	1.39	1	0	0	0	0	Cytoplasmic
45	1	0.23	0	0	0	0	0	

52	0	0.07	1	0	0	0	0	Nucleolar
61	1	0.87	1	0	0	0	0	Homogeneous
18	1	4.30	1	1	1			Homogeneous
34	0	0.08	0	0	0	0	0	
57	0	0.22	0	0	0	0	0	
53	1	0.18	0	0	0	0	0	
27	0	0.14	0	0	0	0	0	
64	0	0.15	0	1		1		
26	1	0.22	0	0	0	0	0	
63	1	0.11	0	0	0	0	0	
19	0	0.16	0	0	0	0	0	
6	0	0.09	0	0	0	0	0	
62	1	0.15	1	0	0	0	0	Homogeneous
44	1	0.14	0	0	0	0	0	
48	1	0.10	0	0	0	0	0	
72	0	0.16	0	0	0	0	0	
48	0	0.09	0	0	0	0	0	
73	1	0.20	0	0	0	0	0	
65	0	0.15	0	0	0	0	0	
37	0	0.32	0	0	0	0	0	
28	0	0.14	0	0	0	0	0	
51	0	11.82	1	1	1			Speckled
62	1	0.10	0	0	0	0	0	
41	0	8.16	1	0	0	0	0	Speckled
13	0	6.76	1	1	1			Homogeneous
20	1	0.21	0	0	0	0	0	
43	0	0.13	0	0	0	0	0	
43	0	0.15	0	0	0	0	0	

47	0	0.08	1	0	0	0	0	Homogeneous
53	0	0.15	0	0	0	0	0	
72	0	0.12	1	0	0	0	0	Homogeneous
75	0	0.31	0	0	0	0	0	
55	1	0.20	0	0	0	0	0	
19	0	0.38	0	0	0	0	0	
39	0	1.35	1	1	1			Cytoplasmic
45	1	0.26	0	0	0	0	0	
50	0	0.10	0	1		1		
67	0	0.08	0	0	0	0	0	
41	0	0.08	1	0	0	0	0	Homogeneous
40	0	0.34	0	0	0	0	0	
30	0	0.13	0	0	0	0	0	
49	1	0.18	0	0	0	0	0	
74	1	0.66	0	0	0	0	0	
49	0	0.31	0	0	0	0	0	
32	0	0.43	0	0	0	0	0	
24	0	5.04	1	0	0	0	0	Speckled
39	0	0.12	1	0	0	0	0	Homogeneous
39	1	0.07	0	0	0	0	0	
59	1	0.06	0	0	0	0	0	
57	0	0.14	0	0	0	0	0	
65	1	0.22	0	0	0	0	0	
32	0	0.34	0	0	0	0	0	
58	1	0.17	0	0	0	0	0	
39	1	0.35	0	0	0	0	0	
48	1	0.13	0	0	0	0	0	
49	0	0.07	1	0	0	0	0	Homogeneous

52	0	0.20	0	0	0	0	0	
58	1	0.11	1	0	0	0	0	Cytoplasmic
47	0	0.65	1	1		1		Cytoplasmic
71	1	0.18	0	0	0	0	0	
40	1	0.34	0	0	0	0	0	
60	0	0.09	0	0	0	0	0	
34	0	0.88	0	1		1		
40	1	0.14	0	0	0	0	0	
20	0	0.15	1	0	0	0	0	Homogeneous
57	0	0.21	1	0	0	0	0	Cytoplasmic
62	0	0.12	1	0	0	0	0	Homogeneous
51	0	0.11	0	0	0	0	0	
49	0	0.08	0	0	0	0	0	
13	0	10.14	1	1	1			Homogeneous
60	0	0.13	0	0	0	0	0	
24	0	0.10	0	0	0	0	0	
47	0	1.31	1	0	0	0	0	Homogeneous
50	1	0.23	0	0	0	0	0	
54	0	0.14	0	0	0	0	0	
28	1	0.23	0	0	0	0	0	
45	0	0.13	0	0	0	0	0	
6	1	0.11	0	0	0	0	0	
55	0	9.99	1	0	0	0	0	Homogeneous
15	1	0.14	0	0	0	0	0	
56	1	0.12	0	0	0	0	0	
10	0	0.88	0	0	0	0	0	
51	0	0.18	0	0	0	0	0	
58	0	0.11	0	1		1		

56	0	0.65	0	0	0	0	0	
50	0	0.46	0	0	0	0	0	
78	0	0.22	1	0	0	0	0	Homogeneous
70	0	0.20	0	0	0	0	0	
55	1	0.18	0	0	0	0	0	
69	1	0.11	0	0	0	0	0	
61	0	0.19	0	0	0	0	0	
41	0	0.11	0	0	0	0	0	
89	0	0.20	0	0	0	0	0	
62	1	0.24	0	0	0	0	0	
36	0	0.13	0	0	0	0	0	
60	1	0.16	1	0	0	0	0	Homogeneous
35	0	0.15	0	1		1		
57	0	0.16	1	0	0	0	0	Homogeneous
41	0	0.15	0	0	0	0	0	
38	0	0.19	0	0	0	0	0	
9	1	0.32	0	0	0	0	0	
21	0	17.57	1	1		1		Speckled
48	0	0.08	0	0	0	0	0	
21	1	0.11	0	0	0	0	0	
69	1	0.12	0	0	0	0	0	
64	0	0.09	0	0	0	0	0	
24	1	0.12	0	0	0	0	0	
39	0	0.19	0	0	0	0	0	
38	1	0.14	0	0	0	0	0	
39	0	0.18	1	0	0	0	0	Homogeneous
55	1	0.15	1	0	0	0	0	Homogeneous
43	1	0.08	0	0	0	0	0	

75	0	0.28	1	1		1		Centromere
46	0	0.19	1	0	0	0	0	Homogeneous
71	0	0.18	1	0	0	0	0	Homogeneous
78	0	0.23	1	0	0	0	0	Homogeneous
75	0	0.30	0	0	0	0	0	
18	1	0.14	0	0	0	0	0	
45	1	0.14	0	0	0	0	0	
70	1	0.14	1	0	0	0	0	Homogeneous
59	1	0.09	1	0	0	0	0	Speckled
34	0	0.88	1	1		1		Speckled
35	0	0.08	0	0	0	0	0	
47	1	0.13	0	0	0	0	0	
80	0	0.09	0	0	0	0	0	
74	0	0.16	1	1				Homogeneous
70	0	0.15	1	0	0	0	0	Homogeneous
58	0	12.22	1	1	1			Homogeneous
8	0	0.46	0	0	0	0	0	
47	0	0.10	0	0	0	0	0	
74	0	0.34	1	1		1		Homogeneous
39	0	0.14	0	0	0	0	0	
38	0	0.31	0	0	0	0	0	
56	0	0.14	0	1		1		
57	0	0.27	0	0	0	0	0	
50	0	0.12	0	0	0	0	0	
25	1	0.14	0	0	0	0	0	
17	1	0.25	0	0	0	0	0	
75	0	0.26	0	0	0	0	0	
49	1	1.01	1	0	0	0	0	Cytoplasmic

15	0	0.12	0	0	0	0	0	
57	0	0.37	1	0	0	0	0	Homogeneous
37	0	0.59	0	0	0	0	0	
49	1	0.12	0	0	0	0	0	
61	0	0.12	0	0	0	0	0	
71	1	0.35	0	0	0	0	0	
27	1	0.67	0	0	0	0	0	
39	0	1.47	1	1	1			Cytoplasmic
50	1	0.15	0	0	0	0	0	
16	1	1.34	0	0	0	0	0	
55	1	0.47	1	0	0	0	0	Cytoplasmic
26	0	0.32	0	0	0	0	0	
50	1	0.10	0	0	0	0	0	
57	0	0.33	1	0	0	0	0	Homogeneous
15	1	0.22	0	0	0	0	0	
45	1	10.83	1	0	0	0	0	Speckled
40	0	0.12	0	0	0	0	0	
52	0	0.14	0	0	0	0	0	
53	0	0.08	0	0	0	0	0	
62	0	0.18	0	1		1		
28	0	0.16	0	0	0	0	0	
53	0	0.09	0	1		1		
48	0	0.11	0	0	0	0	0	
38	0	0.63	1	0	0	0	0	Homogeneous
60	1	0.37	1	0	0	0	0	Homogeneous
32	0	0.11	0	0	0	0	0	
69	1	0.16	0	0	0	0	0	
37	0	0.11	0	0	0	0	0	

30	0	0.20	1	0	0	0	0	Speckled
39	1	0.17	0	0	0	0	0	
51	0	0.24	1	0	0	0	0	Homogeneous
55	0	0.16	0	0	0	0	0	
20	1	0.14	0	0	0	0	0	
68	1	2.36	1	0	0	0	0	Cytoplasmic
37	1	0.12	0	0	0	0	0	
34	0	0.15	0	0	0	0	0	
83	1	0.14	0	0	0	0	0	
74	1	0.29	0	0	0	0	0	
59	1	0.13	0	0	0	0	0	
35	1	0.09	0	0	0	0	0	
39	0	0.05	0	1	1			
50	0	0.11	0	0	0	0	0	
14	1	0.13	0	0	0	0	0	
46	1	0.09	0	0	0	0	0	
50	0	0.09	0	0	0	0	0	
42	0	0.27	0	0	0	0	0	
55	1	0.13	0	0	0	0	0	
52	0	0.17	1	1		1		Homogeneous
22	1	0.33	0	0	0	0	0	
51	1	0.17	0	0	0	0	0	
53	0	0.35	0	1		1		
36	0	0.15	1	1		1		Nucleolar
59	0	0.11	0	0	0	0	0	
80	0	0.32	1	0	0	0	0	Homogeneous
58	0	0.16	0	0	0	0	0	
67	0	0.13	0	0	0	0	0	

26	0	0.15	0	0	0	0	0	
73	0	0.69	0	0	0	0	0	
37	0	0.25	0	0	0	0	0	
14	0	9.49	1	1	1			Other
49	0	0.17	0	0	0	0	0	
28	1	0.27	0	0	0	0	0	
28	1	0.25	0	0	0	0	0	
64	0	0.22	0	0	0	0	0	
32	0	0.56	0	0	0	0	0	
18	0	0.33	1	1			1	Speckled
52	1	0.35	1	0	0	0	0	Homogeneous
17	0	4.99	1	0	0	0	0	Homogeneous
56	0	0.15	0	0	0	0	0	
45	0	0.14	0	0	0	0	0	
18	0	17.74	1	1		1		Other
30	0	0.15	0	1		1		
53	0	4.09	1	0	0	0	0	Homogeneous
75	0	0.15	0	0	0	0	0	
58	1	0.12	0	0	0	0	0	
49	0	0.24	0	0	0	0	0	
53	0	0.80	0	0	0	0	0	
50	1	0.22	0	0	0	0	0	
67	1	0.11	0	0	0	0	0	
74	1	0.27	0	0	0	0	0	
58	0	0.11	0	0	0	0	0	
51	0	0.24	0	0	0	0	0	
61	0	0.29	0	0	0	0	0	
46	0	0.04	0	0	0	0	0	

13	1	0.25	0	0	0	0	0	
65	0	0.46	0	0	0	0	0	
45	0	0.14	0	0	0	0	0	
74	1	0.06	0	0	0	0	0	
66	0	0.17	0	0	0	0	0	
77	1	0.21	0	0	0	0	0	
81	0	0.37	0	0	0	0	0	
44	1	0.34	1	0	0	0	0	Cytoplasmic
44	0	0.31	1	1		1		Homogeneous
25	0	3.92	1	1	1			Speckled
36	0	0.20	0	0	0	0	0	
65	0	0.18	0	0	0	0	0	
47	0	0.15	0	0	0	0	0	
54	0	0.64	1	0	0	0	0	Other
30	1	0.14	0	0	0	0	0	
18	1	14.28	1	1		1		Other
53	0	0.06	0	0	0	0	0	
60	1	0.09	0	0	0	0	0	
46	1	0.23	0	1		1		
63	0	0.53	0	0	0	0	0	
40	0	0.13	0	1		1		
49	0	0.18	1	0	0	0	0	Homogeneous
57	0	0.87	0	0	0	0	0	
79	0	12.07	1	1	1			Speckled
54	1	0.09	0	0	0	0	0	
68	1	0.16	0	0	0	0	0	
71	0	0.09	0	0	0	0	0	
24	1	0.75	1	0	0	0	0	Cytoplasmic

28	1	2.84	0	0	0	0	0	
74	0	0.12	0	0	0	0	0	
51	0	0.61	0	0	0	0	0	
52	1	0.23	1	0	0	0	0	Homogeneous
34	1	0.09	0	0	0	0	0	
13	0	10.53	1	1	1			Homogeneous
52	0	0.26	0	0	0	0	0	
61	0	0.16	0	0	0	0	0	
57	0	1.17	0	0	0	0	0	
73	0	0.16	0	0	0	0	0	
46	1	0.12	0	0	0	0	0	
65	1	0.18	0	0	0	0	0	
64	0	0.20	0	0	0	0	0	
41	0	0.07	1	0	0	0	0	Nucleolar
32	1	0.10	0	0	0	0	0	
56	1	0.11	0	0	0	0	0	
72	1	0.41	0	0	0	0	0	
50	1	0.15	0	0	0	0	0	
69	0	0.10	0	0	0	0	0	
51	0	0.09	0	0	0	0	0	
51	1	0.09	0	0	0	0	0	
18	1	0.15	0	0	0	0	0	
24	1	0.07	0	0	0	0	0	
66	1	0.06	0	0	0	0	0	
82	1	0.15	1	0	0	0	0	Cytoplasmic
43	1	0.40	0	0	0	0	0	
14	1	0.13	0	0	0	0	0	
53	0	0.13	0	0	0	0	0	

57	0	0.27	0	0	0	0	0	
61	0	0.12	0	0	0	0	0	
70	0	0.32	0	1		1		
30	1	0.56	0	0	0	0	0	
60	0	1.00	0	1		1		
71	1	0.33	1	0	0	0	0	Homogeneous
26	0	0.09	0	0	0	0	0	
29	0	0.36	0	0	0	0	0	
23	0	0.12	0	0	0	0	0	
32	1	0.15	0	0	0	0	0	
65	1	0.20	0	0	0	0	0	
38	1	0.21	0	0	0	0	0	
51	0	0.16	0	0	0	0	0	
56	0	0.57	1	0	0	0	0	Cytoplasmic
55	0	0.24	0	1		1		
13	0	15.66	1	1	1			Homogeneous
55	0	7.14	0	1	1			
48	0	0.16	0	1		1		
71	0	0.13	1	0	0	0	0	Homogeneous
32	1	0.09	0	1		1		
24	1	0.05	0	0	0	0	0	
70	0	0.09	0	0	0	0	0	
54	0	0.13	1	0	0	0	0	Other
76	1	0.89	1	1		1		Other
50	1	0.09	0	0	0	0	0	
43	0	0.07	0	1			1	
26	0	17.16	1	1	1			Speckled
53	1	0.10	0	0	0	0	0	

65	1	0.10	0	0	0	0	0	
65	1	0.15	0	0	0	0	0	
36	0	0.06	0	0	0	0	0	
54	1	0.27	0	0	0	0	0	
68	0	0.12	0	0	0	0	0	
31	0	0.05	0	0	0	0	0	
51	0	0.14	0	0	0	0	0	
63	1	0.14	0	0	0	0	0	
56	0	0.38	0	0	0	0	0	
64	1	0.14	0	0	0	0	0	
64	0	0.15	0	0	0	0	0	
39	0	0.16	0	0	0	0	0	
38	1	0.11	0	0	0	0	0	
80	1	0.12	0	0	0	0	0	
61	1	0.14	1	0	0	0	0	Homogeneous
18	0	0.36	0	0	0	0	0	
52	1	0.28	1	0	0	0	0	Other
45	0	0.11	0	0	0	0	0	
51	0	0.40	0	0	0	0	0	
50	1	0.23	0	0	0	0	0	
36	0	0.07	0	0	0	0	0	
21	1	0.18	0	0	0	0	0	
44	0	0.28	1	1		1		Homogeneous
52	0	0.23	0	0	0	0	0	
51	0	0.47	0	0	0	0	0	
50	1	1.01	1	0	0	0	0	Nucleolar
49	1	0.12	0	0	0	0	0	
13	0	0.21	0	0	0	0	0	

8	0	0.31	1	0	0	0	0	Speckled
32	0	0.12	0	0	0	0	0	
62	0	0.14	1	0	0	0	0	Other
72	0	0.13	1	0	0	0	0	Homogeneous
21	0	0.13	0	0	0	0	0	
46	0	0.09	1	1		1		Homogeneous
68	1	0.23	0	0	0	0	0	
37	0	1.41	1	1		1		Speckled
65	0	0.11	1	0	0	0	0	Homogeneous
59	0	0.06	0	0	0	0	0	
53	1	0.10	0	1	1			
52	1	0.05	0	0	0	0	0	
14	1	0.06	0	0	0	0	0	
49	0	0.16	0	0	0	0	0	
31	0	0.07	0	0	0	0	0	
53	0	0.21	0	0	0	0	0	
20	1	0.09	0	0	0	0	0	
23	1	0.08	0	0	0	0	0	
42	0	0.15	0	0	0	0	0	
38	0	0.09	0	1		1		
13	0	28.62	1	1	1			Homogeneous
70	0	0.11	1	0	0	0	0	Homogeneous
43	1	0.16	0	0	0	0	0	
71	0	0.08	0	0	0	0	0	
39	1	0.10	0	0	0	0	0	
61	1	0.33	0	0	0	0	0	
70	0	0.47	0	0	0	0	0	
43	0	24.42	1	1	1			Speckled

53	0	0.12	1	0	0	0	0	Homogeneous
51	1	0.09	0	0	0	0	0	
49	1	0.09	0	0	0	0	0	
28	0	0.22	1	0	0	0	0	Homogeneous
51	1	0.08	0	0	0	0	0	
36	1	0.12	0	0	0	0	0	
22	0	0.22	0	0	0	0	0	
44	0	0.18	1	0	0	0	0	Speckled
28	1	0.22	1	0	0	0	0	Nucleolar
32	1	0.12	0	0	0	0	0	
36	0	0.10	1	1		1		Nucleolar
17	0	0.12	0	0	0	0	0	
39	1	0.17	0	0	0	0	0	
54	1	0.09	0	0	0	0	0	
53	0	0.15	0	0	0	0	0	
48	0	0.14	0	0	0	0	0	
63	1	0.56	1	0	0	0	0	Nucleolar
66	1	0.43	0	1		1		
43	0	0.32	0	1		1		
25	1	0.16	0	0	0	0	0	
31	1	0.27	0	0	0	0	0	
19	0	0.57	0	0	0	0	0	
72	0	0.13	1	0	0	0	0	Other
54	1	0.06	0	0	0	0	0	
73	1	0.08	0	0	0	0	0	
14	1	0.18	1	0	0	0	0	Homogeneous
31	0	1.61	1	1			1	Centromere
48	1	0.22	1	0	0	0	0	Homogeneous

31	0	0.24	0	0	0	0	0	
64	1	0.10	0	0	0	0	0	
55	0	0.11	0	1		1		
61	0	0.27	1	0	0	0	0	Nucleolar
37	0	0.15	0	0	0	0	0	
44	0	0.13	1	0	0	0	0	Speckled
58	1	1.10	0	0	0	0	0	
32	0	0.16	0	0	0	0	0	
54	1	0.10	0	0	0	0	0	
66	1	0.19	0	0	0	0	0	
27	1	0.07	0	0	0	0	0	
53	0	0.07	0	0	0	0	0	
56	0	0.09	0	0	0	0	0	
31	0	0.15	0	0	0	0	0	
50	1	0.07	0	0	0	0	0	
76	1	0.25	1	0	0	0	0	Cytoplasmic
57	0	0.46	1	0	0	0	0	Cytoplasmic
28	1	0.20	1	0	0	0	0	Nucleolar
21	1	0.11	0	0	0	0	0	
37	0	0.14	0	1		1		
7	1	0.20	0	0	0	0	0	
59	1	0.09	0	0	0	0	0	
57	1	0.09	0	0	0	0	0	
48	0	0.56	0	1		1		
28	0	0.13	0	1		1		
21	0	0.24	0	0	0	0	0	
67	0	0.19	0	0	0	0	0	
70	1	2.30	1	0	0	0	0	Speckled

58	1	0.79	1	0	0	0	0	Speckled
47	0	0.19	1	0	0	0	0	Homogeneous
53	1	0.44	0	0	0	0	0	
30	0	0.09	0	0	0	0	0	
26	0	0.08	0	0	0	0	0	
58	1	1.44	1	0	0	0	0	Speckled
76	1	0.23	1	0	0	0	0	Cytoplasmic
33	0	0.12	0	0	0	0	0	
48	0	0.35	1	0	0	0	0	Speckled
55	0	0.10	0	0	0	0	0	
79	1	0.12	1	0	0	0	0	Homogeneous
77	0	0.10	0	0	0	0	0	
21	0	0.11	0	0	0	0	0	
47	1	0.10	0	0	0	0	0	
71	0	0.09	0	0	0	0	0	
53	0	0.05	0	0	0	0	0	
51	1	0.11	0	0	0	0	0	
47	0	0.25	0	0	0	0	0	
81	0	0.08	0	0	0	0	0	
55	0	0.20	1	0	0	0	0	Homogeneous
58	1	0.53	1	0	0	0	0	Cytoplasmic
44	1	0.18	1	1		1		Cytoplasmic
26	1	0.15	0	0	0	0	0	
41	1	0.19	0	0	0	0	0	
71	0	0.25	0	0	0	0	0	
55	0	0.19	1	0	0	0	0	Homogeneous
77	0	0.32	0	0	0	0	0	
50	0	0.46	0	0	0	0	0	

65	1	2.43	1	0	0	0	0	Homogeneous
54	0	0.13	1	0	0	0	0	Homogeneous
74	0	0.07	0	0	0	0	0	
51	0	0.06	1	1		1		Homogeneous
38	0	0.05	0	0	0	0	0	
68	1	0.11	1	1	1			Cytoplasmic
51	0	0.06	0	0	0	0	0	
60	0	0.06	1	1		1		Cytoplasmic
67	0	0.07	0	1		1		
57	0	0.28	1	0	0	0	0	Cytoplasmic
82	0	0.21	0	0	0	0	0	
45	0	0.07	0	1		1		
56	0	1.06	0	1			1	
45	1	1.84	0	1		1		
68	0	0.19	0	0	0	0	0	
59	1	0.17	1	0	0	0	0	Homogeneous
82	0	0.12	0	0	0	0	0	
70	1	0.53	1	1		1		Homogeneous
58	1	0.33	1	0	0	0	0	Cytoplasmic
56	0	0.08	0	0	0	0	0	
53	0	0.09	0	0	0	0	0	
33	1	0.11	0	0	0	0	0	
54	0	0.13	0	1			1	
48	0	0.16	1	1				Homogeneous
70	1	2.09	1	0	0	0	0	Speckled
56	1	0.08	0	0	0	0	0	
53	1	0.28	0	0	0	0	0	
32	0	0.18	0	0	0	0	0	

48	0	0.12	0	1		1		
51	0	0.05	1	1		1		Homogeneous
54	0	0.07	0	0	0	0	0	
68	0	0.42	0	0	0	0	0	
33	1	0.07	0	0	0	0	0	
2	1	0.19	0	0	0	0	0	
49	0	0.19	0	1		1		
6	0	0.10	0	0	0	0	0	
52	1	0.07	0	0	0	0	0	
38	0	0.48	0	0	0	0	0	
56	0	0.09	0	0	0	0	0	
54	1	0.29	0	0	0	0	0	
53	1	0.12	0	1		1		
43	0	0.08	0	0	0	0	0	
38	0	0.05	0	0	0	0	0	
53	0	0.28	0	0	0	0	0	
71	0	0.25	0	1		1		
49	0	0.08	0	0	0	0	0	
46	1	0.06	0	0	0	0	0	
52	1	0.79	0	0	0	0	0	
69	1	0.14	1	0	0	0	0	Speckled
49	0	0.13	0	0	0	0	0	
46	1	0.14	0	0	0	0	0	
74	1	1.87	1	0	0	0	0	Speckled
13	0	46.93	1	1	1			Homogeneous
65	1	1.25	0	0	0	0	0	
45	0	0.72	0	0	0	0	0	
13	1	0.23	0	0	0	0	0	

37	0	0.09	0	0	0	0	0	
78	1	0.17	0	0	0	0	0	
58	0	1.00	1	0	0	0	0	Cytoplasmic
52	0	0.12	0	0	0	0	0	
70	0	0.09	0	0	0	0	0	
31	1	0.06	0	0	0	0	0	
49	0	0.11	1	1		1		Homogeneous
56	0	0.05	0	1		1		
54	0	0.15	0	0	0	0	0	
32	1	0.09	1	0	0	0	0	Cytoplasmic
62	0	1.17	1	0	0	0	0	Centromere
71	1	0.05	0	0	0	0	0	
44	1	0.06	0	0	0	0	0	
42	1	0.19	0	0	0	0	0	
52	0	0.10	1	0	0	0	0	Homogeneous
51	1	0.19	0	0	0	0	0	
51	0	0.06	0	1			1	
67	0	0.04	1	0	0	0	0	Homogeneous
53	0	0.08	1	1		1		Homogeneous
56	1	0.04	0	0	0	0	0	
36	0	0.12	0	0	0	0	0	
32	1	0.10	1	0	0	0	0	Cytoplasmic
45	1	0.09	0	0	0	0	0	
40	0	0.40	0	0	0	0	0	
68	1	0.51	0	1		1		
38	0	0.11	0	0	0	0	0	
36	0	0.06	0	0	0	0	0	
62	0	1.00	1	0	0	0	0	Centromere

62	0	0.11	1	0	0	0	0	Homogeneous
52	1	0.11	0	1		1		
45	0	0.36	1	1		1		Cytoplasmic
63	1	0.20	1	0	0	0	0	Cytoplasmic
47	0	1.72	0	0	0	0	0	
48	0	0.13	0	0	0	0	0	
28	1	0.28	1	0	0	0	0	Speckled
69	0	0.08	0	0	0	0	0	
34	1	0.23	0	0	0	0	0	
53	1	0.32	0	0	0	0	0	
44	1	0.16	0	0	0	0	0	
75	1	0.10	0	1		1		
73	0	0.14	0	0	0	0	0	
45	0	0.18	1	0	0	0	0	Homogeneous
62	1	0.12	0	0	0	0	0	
9	0	0.12	1	0	0	0	0	Homogeneous
2	1	0.12	0	0	0	0	0	
16	1	0.05	0	0	0	0	0	
33	1	0.13	0	0	0	0	0	
70	0	0.22	0	0	0	0	0	
69	0	1.30	1	0	0	0	0	Centromere
12	1	0.18	0	0	0	0	0	
49	1	0.16	0	0	0	0	0	
65	1	1.40	1	0	0	0	0	Homogeneous
67	0	0.05	1	0	0	0	0	Homogeneous
41	0	0.12	1	0	0	0	0	Speckled
52	0	0.11	1	0	0	0	0	Homogeneous
49	0	0.04	0	0	0	0	0	

46	0	0.84	0	0	0	0	0	
26	0	0.08	0	0	0	0	0	
26	1	0.12	0	0	0	0	0	
53	0	0.09	1	1		1		Homogeneous
60	1	0.09	0	0	0	0	0	
49	0	0.20	1	1		1		Homogeneous
53	1	0.06	0	0	0	0	0	
8	0	0.23	1	1		1		Homogeneous
49	0	1.14	0	0	0	0	0	
48	1	0.44	1	0	0	0	0	Cytoplasmic
13	0	0.08	0	0	0	0	0	
49	0	0.10	0	0	0	0	0	
50	0	0.13	1	0	0	0	0	Nucleolar
60	1	0.31	0	0	0	0	0	
26	0	0.28	0	0	0	0	0	
52	0	0.09	1	0	0	0	0	Other
68	0	2.88	1	0	0	0	0	Homogeneous
55	1	0.13	1	0	0	0	0	Cytoplasmic
60	0	0.13	0	0	0	0	0	
30	0	0.06	0	0	0	0	0	
38	0	0.07	0	0	0	0	0	
59	0	0.12	1	0	0	0	0	Homogeneous
50	1	1.30	1	0	0	0	0	Cytoplasmic
35	0	0.08	0	0	0	0	0	
78	0	0.11	0	0	0	0	0	
31	0	0.16	0	0	0	0	0	
59	1	0.12	0	0	0	0	0	
31	1	0.08	0	1		1		

71	0	0.08	0	0	0	0	0	
59	0	0.08	0	1		1		
24	1	0.08	0	0	0	0	0	
48	1	0.09	0	0	0	0	0	
69	0	0.08	0	0	0	0	0	
69	0	0.09	0	0	0	0	0	
55	1	0.07	0	0	0	0	0	
33	1	0.10	0	0	0	0	0	
31	0	0.58	0	0	0	0	0	
45	0	0.23	1	0	0	0	0	Other
20	0	0.17	0	0	0	0	0	
59	1	0.22	0	0	0	0	0	
45	1	0.11	0	0	0	0	0	
45	0	0.10	0	0	0	0	0	
49	0	0.09	0	0	0	0	0	
28	0	11.83	1	0	0	0	0	Speckled
34	0	0.80	0	0	0	0	0	
17	0	0.30	0	0	0	0	0	
56	0	0.09	0	0	0	0	0	
63	0	0.12	1	0	0	0	0	Nucleolar
65	0	0.04	0	0	0	0	0	
53	1	0.04	0	1		1		
61	0	0.16	0	0	0	0	0	
75	1	0.10	0	0	0	0	0	
73	0	0.12	0	0	0	0	0	
25	0	0.55	0	0	0	0	0	
51	0	0.04	0	0	0	0	0	
26	0	0.09	0	0	0	0	0	

52	0	0.07	0	1		1		
38	0	0.08	0	0	0	0	0	
60	1	0.09	0	0	0	0	0	
52	1	0.30	0	0	0	0	0	
79	0	0.04	0	0	0	0	0	
29	1	0.09	0	0	0	0	0	
16	1	0.06	0	0	0	0	0	
10	1	0.47	1	0	0	0	0	Homogeneous
9	1	0.14	0	0	0	0	0	
27	0	0.16	1	0	0	0	0	Cytoplasmic
14	0	6.59	1	1	1			Other
27	0	0.16	1	0	0	0	0	Speckled
38	0	0.12	0	0	0	0	0	
32	1	0.11	0	0	0	0	0	
54	0	0.18	0	0	0	0	0	
31	0	1.14	1	1			1	Centromere
73	1	0.10	0	0	0	0	0	
59	0	0.30	1	0	0	0	0	Homogeneous
69	0	0.35	0	0	0	0	0	
64	1	0.05	0	0	0	0	0	
62	0	0.05	0	0	0	0	0	
41	0	1.36	0	0	0	0	0	
13	1	0.17	1	0	0	0	0	Homogeneous
27	0	0.21	0	0	0	0	0	
46	1	0.33	0	0	0	0	0	
24	1	0.16	0	0	0	0	0	
64	0	0.08	0	0	0	0	0	
18	0	0.25	1	1			1	Speckled

62	0	0.11	0	0	0	0	0	
32	1	0.23	0	0	0	0	0	
66	1	0.59	1	0	0	0	0	Other
58	1	0.37	0	0	0	0	0	
60	1	0.13	0	0	0	0	0	
14	1	0.12	0	0	0	0	0	
84	0	0.05	0	0	0	0	0	
63	0	0.09	0	0	0	0	0	
54	0	0.03	0	0	0	0	0	
51	1	0.62	0	0	0	0	0	
15	1	0.08	0	1		1		
70	0	0.06	0	0	0	0	0	
32	0	0.11	1	0	0	0	0	Other
70	1	0.24	0	0	0	0	0	
30	0	0.16	1	0	0	0	0	Homogeneous
49	1	0.41	0	0	0	0	0	
45	0	2.59	1	1	1			Homogeneous
84	1	0.10	1	0	0	0	0	Homogeneous
60	0	0.13	0	1		1		
44	0	0.39	1	0	0	0	0	Speckled
14	0	0.07	0	0	0	0	0	
47	0	0.09	0	0	0	0	0	
57	0	0.43	0	0	0	0	0	
79	1	0.23	0	0	0	0	0	
40	0	0.10	1	0	0	0	0	Cytoplasmic
25	0	0.08	1	0	0	0	0	Homogeneous
55	1	0.11	0	1		1		
52	1	1.11	1	0	0	0	0	Homogeneous

35	1	0.06	0	0	0	0	0	
12	1	0.19	0	0	0	0	0	
70	1	0.21	0	0	0	0	0	
35	1	0.07	0	0	0	0	0	
54	1	0.79	0	0	0	0	0	
63	1	0.06	0	0	0	0	0	
55	1	0.14	0	0	0	0	0	
55	0	0.05	0	0	0	0	0	
64	0	0.10	1	0	0	0	0	Speckled
19	0	0.13	0	0	0	0	0	
72	0	0.20	0	0	0	0	0	
52	0	0.28	1	0	0	0	0	Cytoplasmic
31	0	0.11	0	0	0	0	0	
47	0	0.11	0	0	0	0	0	
41	0	10.12	1	1	1			Speckled
9	0	0.05	0	0	0	0	0	
40	0	0.08	0	0	0	0	0	
46	1	0.08	0	0	0	0	0	
40	0	0.10	0	0	0	0	0	
39	0	1.45	1	1			1	Other
44	0	0.22	1	0	0	0	0	Speckled
48	0	0.48	1	0	0	0	0	Speckled
50	0	0.26	1	1			1	Other
29	1	0.73	1	1		1		Homogeneous
28	0	0.06	0	0	0	0	0	
49	1	0.30	1	0	0	0	0	Nucleolar
63	1	0.28	0	0	0	0	0	
67	0	0.19	0	0	0	0	0	

27	0	0.12	0	0	0	0	0	
23	1	0.04	1	0	0	0	0	Homogeneous
58	0	10.12	1	1	1			Homogeneous
44	0	0.05	1	0	0	0	0	Homogeneous
52	0	0.24	1	0	0	0	0	Nucleolar
62	1	0.07	0	0	0	0	0	
52	1	0.12	0	0	0	0	0	
84	1	0.11	1	0	0	0	0	Homogeneous
44	0	0.03	1	0	0	0	0	Homogeneous
24	0	0.11	0	0	0	0	0	
57	0	0.15	0	0	0	0	0	
56	0	0.10	0	0	0	0	0	
53	0	0.10	0	1		1		
47	1	0.25	0	0	0	0	0	
43	0	0.19	1	1			1	Homogeneous
73	0	0.08	0	0	0	0	0	
46	1	0.03	0	1		1		
53	0	0.14	0	0	0	0	0	
52	0	0.08	0	0	0	0	0	
50	0	0.06	0	0	0	0	0	
86	0	0.05	0	0	0	0	0	
67	1	0.06	0	0	0	0	0	
35	0	0.07	0	0	0	0	0	
47	0	0.10	0	0	0	0	0	
63	0	0.31	0	0	0	0	0	
42	1	0.10	0	0	0	0	0	
21	1	0.08	0	0	0	0	0	
44	0	0.16	0	0	0	0	0	

29	1	0.61	1	1		1		Homogeneous
68	1	1.44	1	0	0	0	0	Homogeneous
66	0	0.21	0	0	0	0	0	
66	0	0.09	0	0	0	0	0	
17	0	0.17	1	1			1	Homogeneous
59	0	0.05	0	0	0	0	0	
25	0	0.07	0	0	0	0	0	
60	1	0.11	0	0	0	0	0	
48	1	0.87	1	0	0	0	0	Cytoplasmic
68	0	2.45	1	0	0	0	0	Homogeneous
43	0	0.08	0	0	0	0	0	
37	0	0.07	0	0	0	0	0	
42	0	1.88	1	1		1		Speckled
21	0	0.09	0	0	0	0	0	
52	0	2.04	1	0	0	0	0	Cytoplasmic
72	0	0.11	0	0	0	0	0	
66	1	0.11	0	0	0	0	0	
79	1	0.09	0	0	0	0	0	
60	1	0.29	0	0	0	0	0	
51	0	3.33	0	0	0	0	0	
55	0	0.11	0	0	0	0	0	
57	1	0.11	0	0	0	0	0	
77	1	0.05	0	0	0	0	0	
41	0	0.12	0	0	0	0	0	
43	0	0.21	0	1		1		
36	1	0.51	1	1		1		Nucleolar
56	1	0.22	0	0	0	0	0	
17	1	0.08	0	0	0	0	0	

43	0	0.15	0	0	0	0	0	
61	1	0.14	0	0	0	0	0	
58	1	0.11	0	0	0	0	0	
29	1	0.17	0	0	0	0	0	
27	1	0.15	0	0	0	0	0	
53	0	0.22	1	0	0	0	0	Speckled
67	0	0.10	0	0	0	0	0	
54	0	0.04	0	0	0	0	0	
53	1	0.20	0	1		1		
76	1	0.88	1	0	0	0	0	Homogeneous
44	1	0.01	0	0	0	0	0	
39	0	1.84	1	1	1			Cytoplasmic
68	1	0.05	0	0	0	0	0	
20	1	0.06	0	0	0	0	0	
35	0	0.13	0	0	0	0	0	
32	0	0.09	0	0	0	0	0	
19	0	0.34	0	0	0	0	0	
47	1	0.13	0	0	0	0	0	
51	1	0.47	1	0	0	0	0	Cytoplasmic
62	1	0.48	0	0	0	0	0	
65	1	0.15	0	0	0	0	0	
43	1	1.18	0	0	0	0	0	
60	1	0.06	0	0	0	0	0	
41	0	0.33	1	0	0	0	0	Cytoplasmic
53	0	0.11	1	0	0	0	0	Speckled
41	0	0.09	1	0	0	0	0	Other
8	0	0.08	1	0	0	0	0	Homogeneous
7	1	0.14	0	0	0	0	0	

39	0	5.76	1	1	1			Homogeneous
25	0	0.16	0	0	0	0	0	
31	1	0.07	0	0	0	0	0	
72	1	0.03	0	0	0	0	0	
38	0	0.13	0	0	0	0	0	
49	1	0.08	0	0	0	0	0	
12	1	0.17	0	0	0	0	0	
78	1	0.08	0	0	0	0	0	
60	0	0.09	0	0	0	0	0	
36	1	0.29	0	0	0	0	0	
43	0	0.22	1	1			1	Homogeneous
19	1	0.10	0	0	0	0	0	
62	0	0.26	0	0	0	0	0	
64	1	0.12	0	0	0	0	0	
22	1	0.68	0	0	0	0	0	
18	1	0.08	0	0	0	0	0	
63	1	0.25	1	0	0	0	0	Homogeneous
55	1	0.41	1	0	0	0	0	Cytoplasmic
80	0	0.06	0	0	0	0	0	
61	1	0.12	0	0	0	0	0	
53	0	1.12	0	0	0	0	0	
54	1	0.09	0	0	0	0	0	
36	0	0.19	0	0	0	0	0	
43	0	0.09	1	0	0	0	0	Cytoplasmic
10	1	0.13	0	0	0	0	0	
53	1	0.18	1	1		1		Speckled
77	0	0.06	0	0	0	0	0	
17	1	0.17	0	0	0	0	0	

26	0	0.22	0	0	0	0	0	
63	0	0.10	0	0	0	0	0	
27	0	0.10	0	0	0	0	0	
62	0	0.09	0	0	0	0	0	
39	1	0.06	0	0	0	0	0	
39	0	2.25	1	1	1			Cytoplasmic
71	0	0.15	1	0	0	0	0	Homogeneous
21	1	0.26	0	0	0	0	0	
62	0	0.08	0	0	0	0	0	
52	0	0.16	0	1		1		
36	1	0.32	1	1		1		Cytoplasmic
58	1	0.04	0	0	0	0	0	
41	0	0.13	0	0	0	0	0	
28	1	0.14	1	0	0	0	0	Cytoplasmic
44	1	0.08	0	0	0	0	0	
73	0	0.28	1	0	0	0	0	Cytoplasmic
51	0	0.13	0	0	0	0	0	
39	0	0.06	1	0	0	0	0	Speckled
14	0	8.46	1	1	1			Other
13	0	24.81	1	1	1			Homogeneous
62	1	0.09	1	0	0	0	0	Speckled
79	0	4.71	1	0	0	0	0	Speckled
55	0	0.36	0	0	0	0	0	
29	1	0.20	0	0	0	0	0	
42	0	0.09	0	0	0	0	0	
19	0	0.21	1	0	0	0	0	Other
30	0	0.13	1	1		1		Nucleolar
50	1	0.34	0	0	0	0	0	

50	1	0.14	0	0	0	0	0	
54	1	0.12	0	0	0	0	0	
48	0	0.09	0	0	0	0	0	
25	0	0.18	0	0	0	0	0	
46	1	0.10	0	0	0	0	0	
27	0	0.11	1	0	0	0	0	Speckled
53	1	0.22	1	1		1		Speckled
71	1	0.06	0	0	0	0	0	
7	0	0.20	1	0	0	0	0	Homogeneous
56	1	1.84	0	0	0	0	0	
49	0	0.11	1	1		1		Homogeneous
37	0	0.27	1	0	0	0	0	Other
37	0	2.42	1	0	0	0	0	Other
59	1	0.08	0	0	0	0	0	
20	0	0.20	0	0	0	0	0	
31	1	0.14	0	0	0	0	0	
39	0	0.07	1	0	0	0	0	Speckled
58	1	0.07	0	0	0	0	0	
68	1	0.18	0	0	0	0	0	
61	0	0.10	1	1		1		Other
70	0	0.10	0	0	0	0	0	
38	0	0.14	1	0	0	0	0	Homogeneous
53	0	0.12	1	0	0	0	0	Speckled
47	0	0.26	0	0	0	0	0	
3	1	0.22	0	0	0	0	0	
66	1	0.11	0	0	0	0	0	
42	1	0.15	0	0	0	0	0	
47	1	0.09	0	0	0	0	0	

37	1	0.08	0	0	0	0	0	
48	0	0.14	0	0	0	0	0	
46	0	0.09	0	0	0	0	0	
53	1	0.17	0	0	0	0	0	
68	1	0.43	1	0	0	0	0	Homogeneous
67	1	0.14	1	0	0	0	0	Speckled
65	0	0.10	1	0	0	0	0	Homogeneous
64	0	0.12	1	0	0	0	0	Homogeneous
45	1	0.32	0	0	0	0	0	
50	0	0.09	1	0	0	0	0	Homogeneous
31	1	0.14	0	0	0	0	0	
73	1	0.32	1	0	0	0	0	Cytoplasmic
12	0	0.12	0	0	0	0	0	
33	0	0.11	0	0	0	0	0	
10	1	0.13	0	0	0	0	0	
14	0	5.59	1	1	1			Homogeneous
49	0	2.33	1	0	0	0	0	Cytoplasmic
85	1	0.46	1	0	0	0	0	Other
78	1	0.14	0	0	0	0	0	
54	0	0.17	1	1			1	Other
69	0	0.32	0	0	0	0	0	
64	1	0.19	0	0	0	0	0	
51	0	0.09	1	0	0	0	0	Other
44	0	0.15	0	0	0	0	0	
78	0	0.07	0	0	0	0	0	
56	1	0.21	0	0	0	0	0	
63	0	0.09	0	0	0	0	0	
49	1	0.51	0	0	0	0	0	

1	0	0.22	0	0	0	0	0	
69	1	0.16	1	0	0	0	0	Cytoplasmic
39	0	0.34	0	0	0	0	0	
53	0	0.21	1	0	0	0	0	Speckled
47	0	1.73	1	1		1		Homogeneous
76	1	0.41	1	1		1		Homogeneous
81	1	0.26	0	0	0	0	0	
44	0	2.90	1	0	0	0	0	Other
16	1	0.11	0	0	0	0	0	
19	1	0.19	0	0	0	0	0	
32	1	0.09	0	0	0	0	0	
42	1	0.12	0	0	0	0	0	
18	1	0.18	0	0	0	0	0	
44	0	0.10	0	0	0	0	0	
76	0	0.12	0	0	0	0	0	
26	1	0.10	1	0	0	0	0	Homogeneous
43	0	1.18	0	0	0	0	0	
67	0	0.26	0	0	0	0	0	
54	1	0.16	0	0	0	0	0	
67	1	0.12	0	1		1		
16	0	0.12	0	0	0	0	0	
74	0	0.17	1	0	0	0	0	Homogeneous
55	0	0.14	1	0	0	0	0	Other
49	1	0.06	0	0	0	0	0	
64	1	0.14	1	0	0	0	0	Speckled
71	1	0.11	0	0	0	0	0	
23	0	0.06	0	0	0	0	0	
51	0	0.16	0	0	0	0	0	

76	1	0.22	0	0	0	0	0	
41	0	5.10	1	1			1	Speckled
44	0	5.40	1	1	1			Speckled

IIF, indirect immunofluorescence; RA, rheumatoid arthritis; SLE, systemic lupus erythematosus; MCT, mixed connective tissue disease.

[a] Positive results and male were expressed as 1 and negative results and female were expressed as 0.
