# Supplementary material for: Evaluation of an automated connective tissue disease screening assay in Korean patients with systemic rheumatic diseases
Source: PLoS One. 2017 Mar 8;12(3):e0173597. doi: 10.1371/journal.pone.0173597 (PMC5342238; doi:10.1371/journal.pone.0173597)
Supplement: S1 Table — (DOC) [file pone.0173597.s002.doc]

**S1 Table. The 17 antigens^a^ coated with the EliA CTD screen.**

| Human recombinant antigens | U1RNP 70 | SS-A/Ro 60 kDa | Jo-1 | PM-Scl |
| --- | --- | --- | --- | --- |
|  | U1RNP A | SS-A/Ro 52 kDa | Fibrillarin | PCNA |
|  | U1RNP C | SS-B/La | RNA pol III | Mi-2 protein |
|  | Centromere B | Scl-70 | Rib-P | Sm protein |
| Native purified antigen | DNA | | | |

^a^ The antigens were indicated in the instructions provided by the manufacturer.
