# Supplementary material for: Evaluation of an automated connective tissue disease screening assay in Korean patients with systemic rheumatic diseases
Source: PLoS One. 2017 Mar 8;12(3):e0173597. doi: 10.1371/journal.pone.0173597 (PMC5342238; doi:10.1371/journal.pone.0173597)
Supplement: S2 Table — (DOC) [file pone.0173597.s003.doc]

**S2 Table. The *P* values^a^ among variables of subgroups, including RA, SLE, MCT, and control.**

| Subgroup | Sex | Age | IIF | IIF pattern | CTD screen |
| --- | --- | --- | --- | --- | --- |
| RA vs. control | 0.0028 | 1.0000 | <0.0001 | 0.9896 | 0.2371 |
| SLE vs. control | <0.0001 | <0.0001 | <0.0001 | 0.5589 | <0.0001 |
| MCT vs. control | <0.0001 | 0.0134 | <0.0001 | 0.0002 | <0.0001 |

RA, rheumatoid arthritis; SLE, systemic lupus erythematosus; MCT, mixed connective tissue disease.

^a^ The *P* values were calculated using the Kruskal-Wallis test and Chi-square test with Bonferroni correction to compensate for alpha statistical errors.
