# Supplementary material for: Evaluation of an automated connective tissue disease screening assay in Korean patients with systemic rheumatic diseases
Source: PLoS One. 2017 Mar 8;12(3):e0173597. doi: 10.1371/journal.pone.0173597 (PMC5342238; doi:10.1371/journal.pone.0173597)
Supplement: S3 Table — (DOC) [file pone.0173597.s004.doc]

**S3 Table. The study population characteristics and the results of IIF and CTD screen in the sex- and age-matched controls.**

| Sex group | Parameters | Total disease | Total disease without RA | Control | *P* value^a^ | *P* value^b^ |
| --- | --- | --- | --- | --- | --- | --- |
| Male (n = 450)^c^ | Age (years)^d^ | 52.5 (36.0-63.1) | 53.0 (23.8-65.5) | 50.5 (33.0-62.0) | 0.8457 | 0.9981 |
|  | IIF (No.) positive | 13 | 2 | 83 | 0.0163 | 0.2103 |
|  | IIF (No.) negative | 19 | 1 | 335 |  |  |
|  | CTD screen (ratio)^d^ | 0.2 (0.1-0.5) | 0.1 (0.1-3.6) | 0.1 (0.1-0.2) | 0.2172 | 0.8378 |
| Female (n = 643)^c^ | Age (years)^d^ | 44.0 (33.8-53.0) | 39.0 (18.0-47.5) | 50.0 (37.7-62.0) | 0.0001 | <0.0001 |
|  | IIF (No.) positive | 87 | 53 | 144 | <0.0001 | <0.0001 |
|  | IIF (No.) negative | 43 | 6 | 369 |  |  |
|  | CTD screen (ratio)^d^ | 0.3 (0.1-4.2) | 4.2 (1.1-10.1) | 0.1 (0.1-0.2) | <0.0001 | <0.0001 |
| Age group^e^ | No. of females/No. of total, % | 47/56, 83.9 | 30/31, 96.8 | 148/281, 52.7 | <0.0001 | <0.0001 |
| <40 (n = 337)^c^ | IIF (No.) positive | 45 | 30 | 46 | <0.0001 | <0.0001 |
|  | IIF (No.) negative | 11 | 1 | 235 |  |  |
|  | CTD screen (ratio)^d^ | 1.4 (0.2-6.7) | 4.2 (1.5-10.0) | 0.1 (0.1-0.2) | <0.0001 | <0.0001 |
| 40-50 (n = 206)^c^ | No. of females/No. of total, % | 37/43, 86.0 | 15/15, 100.0 | 101/163, 62.0 | 0.0033 | 0.0023 |
|  | IIF (No.) positive | 29 | 14 | 43 | <0.0001 | <0.0001 |
|  | IIF (No.) negative | 14 | 1 | 120 |  |  |
|  | CTD screen (ratio)^d^ | 0.3 (0.2-2.4) | 4.7 (0.4-5.9) | 0.1 (0.1-0.3) | <0.0001 | <0.0001 |
| 50-60 (n = 253)^c^ | No. of females/No. of total, % | 32/41, 78.0 | 11/12, 91.7 | 114/212, 53.8 | 0.0055 | 0.0163 |
|  | IIF (No.) positive | 15 | 7 | 57 | 0.2850 | 0.0524 |
|  | IIF (No.) negative | 26 | 5 | 155 |  |  |
|  | CTD screen (ratio)^d^ | 0.1 (0.1-0.2) | 1.2 (0.1-10.2) | 0.1 (0.1-0.3) | 0.3669 | 0.0038 |
| >60 (n = 297)^c^ | No. of females/No. of total, % | 14/22, 63.6 | 3/4, 75.0 | 150/275, 54.5 | 0.5508 | 0.7746 |
|  | IIF (No.) positive | 11 | 4 | 81 | 0.0841 | 0.0164 |
|  | IIF (No.) negative | 11 | 0 | 194 |  |  |
|  | CTD screen (ratio)^d^ | 0.1 (0.1-0.3) | 6.1 (0.1-13.2) | 0.3 (0.1-0.5) | 0.0152 | 0.1087 |
| Sex- and age-matched group^f^ | Age (years)^d^ | 45.0 (34.0-53.1) | 39.5 (18.0-50.1) vs. 39.5 (19.0-50.1)^g^ | 45.0 (34.0-53.1) | 0.9343 | 0.7715 |
|  | No. of females/No. of total, % | 130/162, 80.2 | 59/62, 95.2 vs. 59/62, 95.2^g^ | 130/162, 80.2 | 1 | 1 |
|  | IIF (No.) positive | 100 | 55 vs. 10^g^ | 33 | <0.0001 | <0.0001 |
|  | IIF (No.) negative | 62 | 7 vs. 52^g^ | 129 |  |  |
|  | CTD screen (ratio)^d^ | 0.3 (0.1-2.3) | 4.1 (0.3-10.1) vs. 0.1 (0.1-0.2)^g^ | 0.1 (0.1-0.2) | <0.0001 | <0.0001 |

IIF, indirect immunofluorescence; RA, rheumatoid arthritis.

^a^ Fisher's exact test for nominal variables and Mann-Whitney U test for continuous variables of the total disease vs. control.

^b^ Fisher's exact test for nominal variables and Mann-Whitney U test for continuous variables of the total disease without RA vs. control.

^c^ The numbers of sex and age group in parenthesis are the sum of the numbers of the total disease group and control group.

^d^ Data are expressed as median (1st to 3rd quartiles).

^e^ Age group was classified depending on the distribution (1st quartile, 36.0; median, 50.0; 3rd quartile, 61.0).

^f^ The each number of the total disease group and matched control group was 162, and that of the total disease group without RA and matched control group was 62.

^g^ Data are expressed as values of total disease without RA group vs. those of matched control group.
